# Effect of a Single Application of CPP-ACPF Varnish on the Prevention of Erosive Tooth Wear: An AAS, AFM and SMH Study

**DOI:** 10.3290/j.ohpd.a43365

**Published:** 2020-07-04

**Authors:** Berna Gokkaya, Nil Ozbek, Zeliha Guler, Suleyman Akman, A. Sezai Sarac, Betul Kargul

**Affiliations:** ^a^ Pediatric Dentist, Clinic of Pediatric Dentistry, Bahcelievler Oral and Dental Health Hospital, Turkey. Conducted study and wrote the manuscript.; ^b^ Assistant Professor, Faculty of Arts and Sciences, Department of Chemistry, Istanbul Technical University, Turkey. Did all laboratory works for atomic absorption spectroscopy (AAS).; ^c^ Assistant Professor, Department of Nanoscience and Nanoengineering, Istanbul Technical University, Turkey. Did all laboratory works for atomic force microscopy (AFM).; ^d^ Professor, Faculty of Arts and Sciences, Department of Chemistry, Istanbul Technical University, Turkey. Analysed AAS data.; ^e^ Professor, Department of Nanoscience and Nanoengineering, Istanbul Technical University, Turkey; Department of Chemistry and Polymer Science and Technology, Istanbul Technical University, Turkey. Analysed AFM data.; ^f^ Professor, Department of Pediatric Dentistry, Marmara University, Turkey. Planned research and study groups, checked the manuscript.

**Keywords:** AFM, AAS, CPP-ACPF, erosion, SMH

## Abstract

**Purpose:**

The aim of this study was to investigate the in vitro effect of casein phosphopeptide–amorphous calcium phosphate (CPP-ACP) and fluoride-containing varnish on prevention enamel erosive tooth wear.

**Materials and Methods:**

A total of 28 enamel samples were prepared from human molars, divided into four groups: CPP-ACPF varnish, TCP-F varnish, NaF varnish, and deionised water. For the remineralisation process stimulated human pooled saliva was used. After treatment, all enamel samples were exposed to 10 ml of Coca Cola. Ca^++^ release was determined by atomic absorption spectroscopy (AAS). The surface topography was evaluated by atomic force microscopy (AFM). Surface microhardness of enamel was analysed and SMHR % (surface microhardness reduction ) was calculated. Data were analysed with repeated measures analysis of variance (ANOVA).

**Results:**

Deionised water demonstrated a statistically significantly higher Ca^+2^ release compared to those of groups NaF > fTCP > CPP-ACPF, respectively (p <0.01). All groups measured for root-mean-square-roughness (R_rms_) showed a statistically significantly difference of 6 × 6 μm^2^ and 12 × 12 μm^2^ enamel area (p <0.05) compared with a negative control group. CPP-ACPF varnish showed rougher surfaces than all remineralisation groups. SMHR % of enamels were as follows: CPP-ACPF < fTCP < NaF < deionised water (p <0.01).

**Conclusion:**

According to the findings of this study; CPP-ACP containing agents have a statistically statistically significant effect on preventing dental erosion. Among these, CPP-ACPF-containing remineralisation agents have the most effect on the remineralisation process.

Dental erosion is a challenge for the 21st century. For many years, erosive tooth wear was a condition of little interest to clinical dental practice, dental research and dental public health. However, perceptions have now changed. First of all, there is no fixed critical pH value concerning dental erosion.^1,10,23,27^ Erosion occurs with low pH; also there is no fixed critical pH value concerning dental erosion.^16^ Usually in relation to erosive solutions, the main focus is extremely concentrated on the hydrogen ion concentration (pH). In this case, the concentration of calcium is the most important factor determining the critical pH, but fluoride and phosphate concentrations, in other components, will also play a role. According to that these concentrations will vary from solution to solution, and they will be different to those found in plaque fluid. The critical pH for enamel will also vary in the case of erosion. In cases where the mineral concentrations are higher than the values found in plaque fluid, the solution will not be able to dissolve tooth mineral even if its pH is below 5.5. As a result, there is no fixed critical pH value concerning dental erosion.

In recent years, different agents for inhibiting erosive tooth wear have been studied, such as casein phosphopeptides with amorphous calcium phosphate complex (CPP-ACP). The CPP-ACP complex may increase the level of calcium and inorganic phosphate ions at the tooth surface, thereby permitting immediate enamel surface remineralisation.^21,24,33^ When placed on the surface of a tooth, CPP-ACP interact with hydrogen ions and form calcium hydrogen phosphate, which releases calcium and phosphate ions; so CPP-ACP therefore aid in remineralisation.^4^ The CPP-ACP complexes readily combine with fluoride ions to form CPP-ACPF. CPP-ACPF provides additional fluoride along with calcium and phosphate ions for remineralisation.^4^ This CPP-ACPF complex, when incorporated into toothpaste, mouth rinses, chewing-gum, varnishes or sprays, is able to join to the dental biofilm and enamel hydroxyapatite, providing a reservoir of bioavailable calcium and phosphate ions. These agents are useful in reversing the erosive demineralisation caused by the contact of acidic food and drinks with teeth.^6^ Therefore it is essential to ensure these innovative materials release bioavailable calcium, phosphate and fluoride ions and protect enamel against acid demineralisation at least as well as, and hopefully significantly better, than the normal fluoride-alone dental varnishes.

The aim of this study was to evaluate the ability of these novel calcium phosphate- and fluoride-containing varnishes to release calcium, phosphate and fluoride ions and to inhibit enamel erosion.

## MATERIALS AND METHODS

Twenty-eight permanent human premolars extracted from 10- to 12-year-old children for orthodontic purposes were used in this study. Before the extraction, the patients were informed about the use of their teeth for research purposes and consent was obtained. Teeth were stored in a 0.1% thymol solution. Enamel samples were cut from each crown using an ISOMET Low Speed Saw cutting machine (Buehler, Lake Bluff, IL, USA). Samples were placed into Teflon moulds measuring 4 × 4 × 2 mm. The samples were embedded in self-cured acrylic resin and surfaces were wet ground using 400, 600, 800 and 1200 grit silicon carbide paper to obtain a smooth flat surface. Each sample was then divided into two halves, with one half of each specimen coated with red nail polish for determination of the enamel area. To mimic the clinical conditions as closely as possible, the study was performed with human extracted teeth and human pooled saliva as a natural remineralisation source.

In this study, stimulated human saliva was collected from healthy donors upon Ethical Committee of Marmara University (protocol #2014–7) approval and informed consent.

### Human Pooled Stimulated Saliva

Paraffin-wax-stimulated saliva from 15 healthy donors was collected into ice-chilled vials (5 ml for every donor), pooled, and centrifuged (4000 Å~ g/4°C/15 min). The supernatant was collected for the experiment and stored at −20°C between experiments. Each defrosted saliva portion was transparent, had a pH of 7.80 ± 0.03 and calcium (Ca^2+^) content of 1.25 mM. Enamel samples were incubated in human saliva for 1 h. Then, 28 samples were prepared and divided into four groups after post-hoc power analysis (n = 7 for each group), which were treated with the dental materials that follow.

In this study, CPP-ACPF varnish (10% CPP-ACP and 22,500 ppm F) (GC Corporation, Tokyo, Japan) and fTCP (TCP and 22,500 ppm F) (3M Oral Care, CA, USA) were evaluated. NaF varnish (22,600 ppm NaF) (Colgate Oral Care, NSW, Australia) was chosen as the positive control, and the negative control group received no treatment.

- Group 1: CPP-ACPF varnish (10% CPP-ACP and 22,500 ppm F)
- Group 2: fTCP (TCP and 22,500 ppm F)
- Group 3: positive control: NaF varnish (22,600 ppm NaF)
- Group 4: negative control: (deionised water)

All of the varnishes were applied to cover specimen surfaces; the treated surfaces were left for 20 s and then cleaned with deionised water.

### Erosion Process

A soft drink (Coca Cola, Coca Cola Company, Milan, Italy) was chosen for the erosion process. All of the specimens were immersed in 10 ml Coca Cola (pH = 2.6) for 10 min at 37°C to simulate the oral environment. All acidic solutions were stored at 4°C and further used for the analysis of calcium release from each sample. The same procedures, parameters, and instruments were used for calcium analysis in the acidic solutions via atomic absorption spectroscopy (AAS).

Samples were removed from the acidic solutions, carefully rinsed with deionised water (20 s), and dried with oil-free air (5 s). Surface morphologies and surface root-mean-square roughness (R_rms_) were investigated using three specimens in each group via an atomic force microscope (Nanosurf EasyScan2, Nanosurf, Liestal, Switzerland) in non-contact mode using Al-coated high resonance frequency silicon tips (Nanosensors NCRL tips, 40 μm width, 225 μm length).

The surface microhardness of after erosion was measured with Wolpert Wilson Micro-Vickers 401MVD (Wolpert Wilson, MA, USA). The surface microhardness of enamel was measured on the surface by means of a Vickers indenter with 200 g of force for 15 s. On the other hand, nail polish was cleaned from the surface, and the surface microhardness at baseline was measured with Vickers. Additionally, the percentage reduction in surface microhardness (SMHR %) was calculated as:

SMHR % = Microhardness (at baseline) – Microhardness (after erosion) × 100 Microhardness (at baseline)

## RESULTS

### AAS Results

Measurement of Ca^2+^ release into acidic solutions for all treatment groups showed statistically significantly lower Ca^2+^ release compare to deionised water (p <0.05) (Table 1). NaF varnish demonstrated higher Ca^2+^ release compared to fTCP varnish and CPP-ACPF varnish (p <0.01) (Table 1). When comparing fTCP varnish, NaF varnish and CPP-ACPF varnish no statistically significant difference was found between these groups (Table 1) (p >0.05).

**Table 1:** Mean and standard deviation (SD) of Ca^2+^ release in all groups

	Ca^2+^
	Mean ± SD
CPP-ACPF varnish	1.76 ± 0.19
fTCP varnish	1.85 ± 0.14
NaF varnish	1.89 ± 0.25
Deionised water	2.91 ± 0.38
P	0.001**

Kruskal–Wallis test, *p <0.05.

### Atomic Force Microscopy (AFM) Results

**Fig 1:**
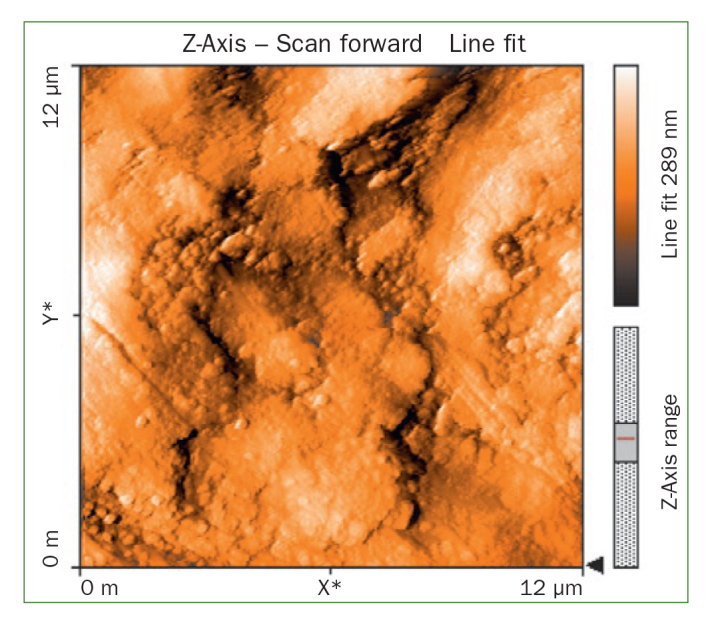
Two-dimensional images of CPP-ACPF varnish.

**Fig 2:**
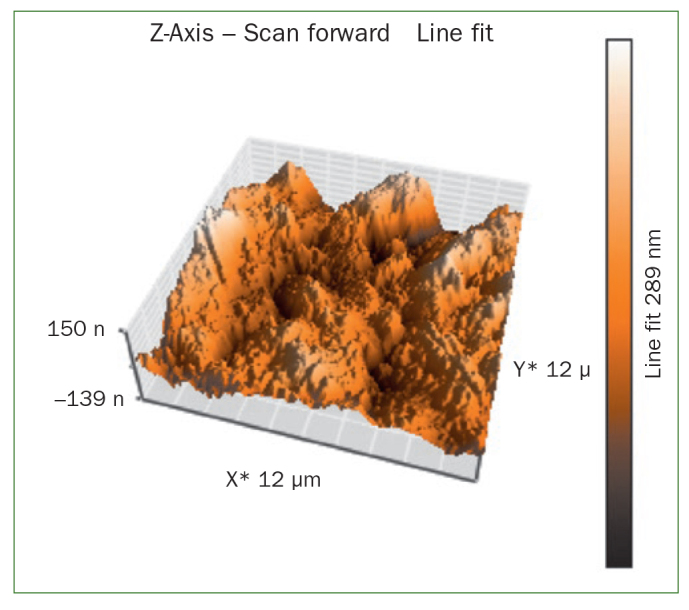
Three-dimensional images of CPP-ACPF varnish.

**Fig 3:**
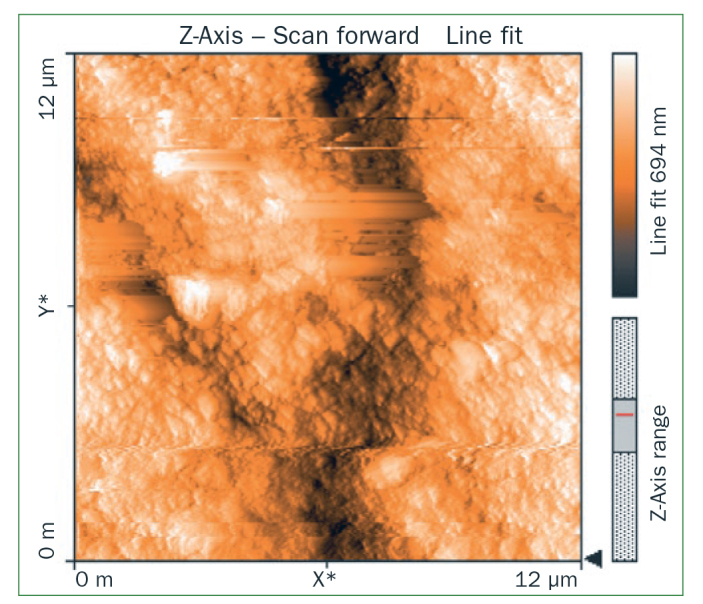
Two-dimensional images of NaF varnish.

**Fig 4:**
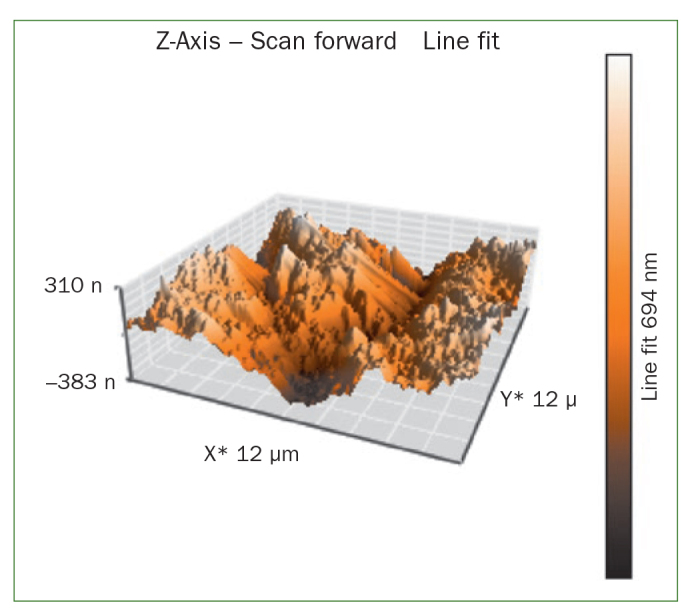
Three-dimensional images of NaF varnish.

**Fig 5:**
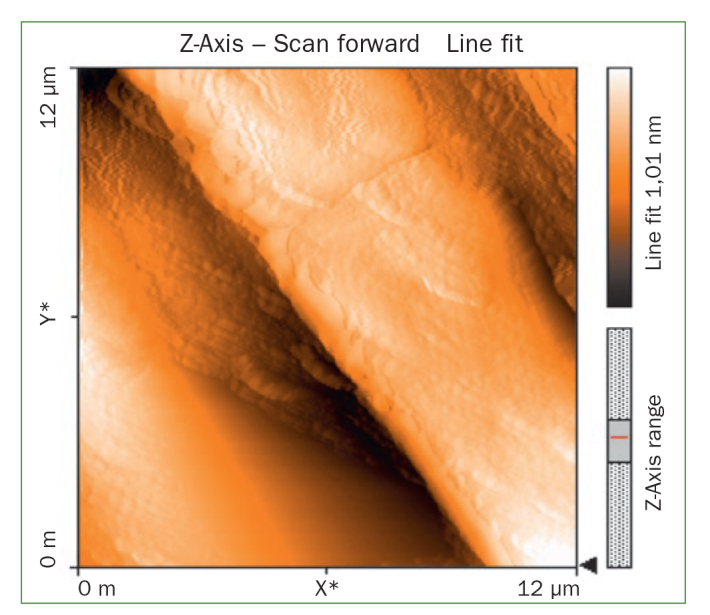
Two-dimensional images of fTCP varnish.

**Fig 6:**
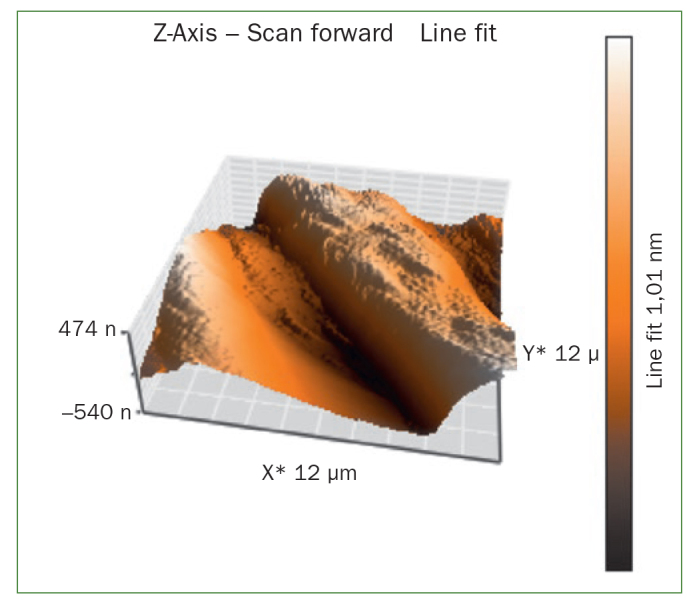
Three-dimensional images of fTCP varnish.

The AFM results show that the use of CPP-ACP offers a statistically significant advantage with respect to the remineralisation of eroded enamel; it can form hydroxyapatite (HA) crystals to repair the enamel prisms and enamel interprisms (Figs 1–4). The nanomechanical properties are also significantly improved during this process as well, and with the extended remineralisation periods, the enamel surface becomes smoother. By contrast, enamel prism and enamel interprism structures became clearly visible on AFM images after acid etching of the smoothed natural enamel surface (Figs 5 and 6).

CPP-ACPF varnish, NaF varnish and fTCP varnish showed statistically significant differences (p <0.05) in mean-square-roughness (R_rms_) values was registered in 6 × 6 μm^2^ and 12 × 12 μm^2^ enamel areas compared with deionised water. CPP-ACPF varnish showed lower R_rms_ values compared with fTCP varnish and NaF varnish in 6 × 6 μm^2^ and 12 × 12 μm^2^ enamel areas (p <0.05) (Table 2).

**Table 2:** Mean±SD and p values of 6 × 6 μm^2^ and 12 × 12 μm^2^ areas with respect to surface roughness (R_rms_)

	R_rms_ (6 × 6 μm^2^)	R_rms_ (12 × 12 μm^2^)
	Mean ± SD	Mean ± SD
CPP-ACPF varnish	33.78 ± 13.39	59.33 ± 19.02
NaF varnish	57.05 ± 14.87	69.68 ± 2.61
fTCP varnish	40.13 ± 28.46	100.83 ± 40.9
Deionised water	147.54 ± 69.15	318.7 ± 140.31
P	0.029 *	0.023 *

Kruskal–Wallis test, *p <0.05.

Remineralisation in the CPP-ACPF varnish, NaF varnish and fTCP varnish groups was significantly better than in the control group (p <0.01), but there was no statistically significant difference between treatment groups (p >0.05) (Table 3).

**Table 3:** Mean ± SD and p values of surface microhardness (SMH) at baseline and after remineralisation and erosion

	Baseline microhardness	Microhardness after remineralisation and erosion	
	Mean ± SD	Mean ± SD	^3^p
CPP-ACPF	316.45 ± 31.93	278.50 ± 21.43	0.00**
fTCP	308.22 ± 19.44	263.56 ± 21.93	0.00**
NaF	315.58 ± 25.32	263.50 ± 18.54	0.00**
Deionised water	315.89 ± 14.79	213.12 ± 33.46	0.00**
P	^1^0.048**	^1^0.000**	

^1^ One-way ANOVA test; ^3^ Paired samples test; ** p <0.01.

### SMH Results

The SMHR % of NaF varnish was higher than the SMHR % of CPP-ACPF varnish (p <0.05). There was no statistically significant difference between CPP-ACPF varnish and fTCP varnish and NaF varnish (p >0.05). By contrast, all treatment groups exhibited a statistically significantly better protective effect compared to the control group (p <0.01) (Table 4).

**Table 4:** Mean ± SD and p values of surface microhardness reduction % (SMHR %)

	SMHR %
	Mean ± SD
CPP-ACPF	15.20 ± 8.39
fTCP	17.64 ± 11.63
NaF	20.42 ± 14.31
Deionised water	51.62 ± 24.89
P	^2^0.000**

^2^ Kruskal–Wallis test; ** p <0.01.

Statistically significantly positive correlations between Ca^2+^ release and R_rms_ % were found (p <0.05) (Table 5). Ca^2+^ release was able to describe the variability of SMHR % up to 27% (p = 0.223).

**Table 5:** Correlation between SMHR % and Ca^2+^ release

	Correlation of SMHR % and Ca^2+^ release	
	r	^1^p
CPP-ACPF varnish	–0.213	0.686
fTCP varnish	–0.395	0.439
NaF varnish	0.177	0.738
Deionised water	–0.429	0.397
All groups	0.350	^1^0.223**

^1^Spearman correlation test; ** p <0.05.

The regression curve between Ca^2+^ release and SMHR % showed a statistically significantly positive slope (b = 18.96) (p = 0.00) (Table 6).

**Table 6:** Regression between Ca^2+^ release and SMHR %: means and p values

	Regression between Ca^2+^ release and SMHR %			
	a**	b**	R^2^	^1^p
CPP-ACPF varnish	30.28	–8.61	0.282	0.278
fTCP varnish	14.01	2.00	0.001	0.961
NaF varnish	41.70	–11.63	0.041	0.702
Deionised water	94.95	14.69	0.058	0.647
All remineralisation agents	–15.49	18.96	0.273	0.00**

**p <0.05; a**, intercept; b**, slope; R^2^, coefficient of determination.

A 12 × 12 μm^2^ enamel area surface roughness (R_rms_) values were used to evaluate the correlation between SMHR % and surface roughness.

A statistically significant positive correlation was found between SMHR % and R_rms_ (p <0.05) (Table 7). Surface roughness effected SMHR % up to 47% (p = 0.037).

**Table 7:** Correlation between SMHR % and surface roughness (R_rms_)

	Correlation between SMHR % and R rms	
	r	^1^p
CPP-ACPF varnish	1.000	0.000**
fTCP varnish	1.000	0.000**
NaF varnish	–1.000	0.000**
Deionised water	–0.500	0.667
All groups	0.524	^1^0.037**

^1^ Spearman correlation test; **p <0.05.

The regression curve between R_rms_ and SMHR % showed a statistically significantly positive slope (b = 0.065) (p = 0.003) (Table 8).

**Table 8:** Regression between R_rms_ and SMHR %: means and p values

	Regression between R_rms_ and SMHR %			
	a**	b**	R^2^	^1^p
CPP-ACPF varnish	–129.0	2.03	1.000	-
fTCP varnish	191.9	–1.40	1.000	-
NaF varnish	17.56	–0.07	1.000	-
Deionised water	22.50	0.03	0.247	0.669
All of groups	11.07	0.065	0.474	0.003**

**p <0.05; a**, intercept; b**, slope; R^2^, coefficient of determination.

There was a positive correlation between Ca^2+^ release and R_rms_, however it was not statistically significant (p >0.05) (Table 9). Ca^2+^ release effected R_rms_ up to 57% (p = 0.163).

**Table 9:** Correlation between Ca^2+^ release and R_rms_: means and p values

	Correlation between Ca^2+^ release and R_rms_	
	r	^1^p
CPP-ACPF varnish	1.000	-
fTCP varnish	-	-
NaF varnish	–1.000	-
Deionised water	1.000	-
All groups	0.366	0.163

^1^Spearman correlation test;**p <0.05.

The regression between Ca^2+^ release and R_rms_ was found to be statistically significant regarding the positive slope (b = 0.003) (p = 0.001) (Table 10).

**Table 10:** Regression between Ca^2+^ release and R_rms_: means and p values

	Regression between Ca^2+^ release and R_rms_			
	a**	b**	^R^2^^	^1^p
CPP-ACPF varnish	–1.153	0.048	1.000	-
fTCP varnish	-	-	-	-
NaF varnish	2.086	–0.005	1.000	-
Deionised water	2.668	0.001	0.995	0.045**
All groups	1.822	0.003	0.574	0.001**

**p <0.05; a**, intercept; b**, slope; R^2^, coefficient of determination.

The regression curve between Ca^2+^ release, R_rms_ and SMHR % showed a statistically significant positive slope (b_1 _= 5.59; b_2 _= 0.049). Ca^2+^ release and R_rms_ effected SMHR % up to 49% (p = 0.012) (Table 11).

**Table 11:** Regression between Ca^2+^ release, R_rms_ and SMHR %, mean and p values

	Regression between Ca^2+^ release, R_rms_ and SMHR %				
a**	b_1_**	b_2_**	R^2^	^1^p
CPP-ACPF varnish	–129	2.03	-	1000	-
fTCP varnish	191.9	–1.4	-	1000	-
NaF varnish	17.56	–0.07	-	1000	-
Deionised water	2752.2	–1023.2	0.843	1000	-
All groups	0.868	5.59	0.049	0.496	0.012**

**p <0.05; a**, intercept; b_1_ **, 1. Slope; b_2_, 2. Slope.

## DISCUSSION

Many strategies have been used to prevent erosion in enamel, such as highly concentrated fluoride applications in the form of oral rinses, gels or varnishes.^9,15,17^ Fluoride varnishes may be more effective because they provide long contact periods between the dental tissues and the fluoride agent, which results in high fluoride uptake and the formation of CaF_2_ deposits that act as fluoride reservoirs.^9,14,18^ The protective effect of sodium fluoride against dental erosion has been shown in previous studies.^5,9,17^ In addition to fluoride, other minerals, such as calcium and phosphate, may be used to enhance the protective/strengthening benefits of fluoride to better address dental erosion.^8^ The use of calcium and phosphate products together with fluoride has been reported to have a synergistic effect,^12,13^ therefore, a sodium fluoride varnish, a sodium fluoride varnish containing tricalcium phosphate, and a sodium fluoride varnish containing CPP-ACP were included among the treatment regimens that were tested.

**Fig 7:**
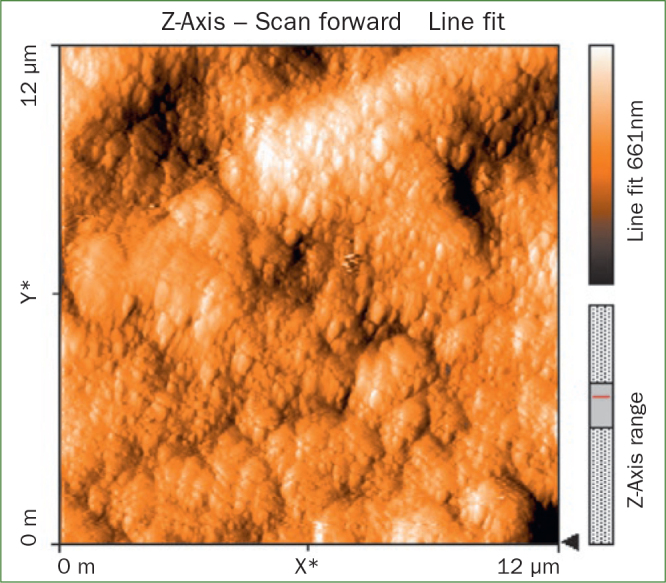
Two-dimensional images of deionised water.

**Fig 8:**
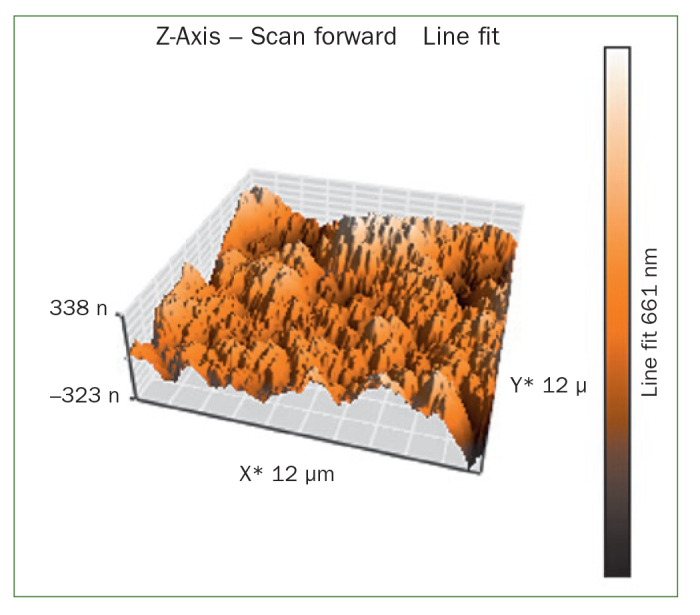
Three-dimensional images of deionised water.

In the present study enamel samples were incubated in human saliva for 1 h and following this procedure, the test varnishes were applied for 20 s each. This procedure is the same as Elkassas et al^7^ and then samples washed with deionised water (see Figs 7 and 8).

Measurements of calcium release into acidic solutions and change of enamel surface roughness are usually applied for the assessment of dental erosion in vitro.^2,30,31,32^ Calcium dissolution typically results in softening of the enamel first, followed by a gradual tissue wear if the calcium loss persists. Calcium ions were released from all varnishes tested. The greatest calcium release was from NaF varnish followed by fTCP and CPP-ACPF varnish. Similar to the results of the calcium release analysis, the microhardness change in the three groups indicated that CPP-ACPF application on enamel was more effective to inhibit the erosion procedure.

CPP-ACP promoted an increase of the surface hardness,^19^ reducing tooth wear^24,25^ and erosion depth.^20^ Calcium and phosphate ions are building blocks for the remineralisation process and are found in saliva. CPP-ACP has been introduced as a supplemental source of calcium and phosphate ions in the oral environment. Amorphous calcium phosphate is biologically active and is able to release calcium and phosphate ions to maintain saturation levels of calcium and phosphate at the tooth surface. It is hypothesised that, in addition to the prevention of erosive demineralisation, CPP-ACP also remineralises eroded enamel and dentine crystals. This hypothesis is supported by an observation that superficial granular structures, probably representing remineralised enamel crystals, were formed on the enamel surface after exposure to a sports drink containing CPP-ACP.^11^ In the present study, NaF group exhibited higher Ca^2+^ release compared to CPP-ACPF and fTCP. Hegde et al^11^ found that CPP-ACPF showed a lower calcium release when compared to fTCP, which was not statistically significant. These results can be attributed to the fact that fluoride in sub-ppm concentrations is effective in promoting mineral deposition and inhibiting mineral dissolution. Shen et al^28^ said that CPP-ACPF varnish also released the highest levels of calcium, phosphate and fluoride ions compared to fTCP, NaF varnish and ACP containing varnish. The previous results of these studies are similar with the finding of this study. Savas et al^26^ evaluated the efficacy of CPP-ACPF varnish for remineralising white spot lesion with four quantitative methods. They found that CPP-ACP-containing fluoride varnish provides remineralisation of incipient carious lesions after a single application and seems suitable for clinical use. In this study, CPP-ACPF varnish were applied with different approaches as preventive treatments using a model simulating oral conditions with natural saliva. Therefore, the authors of the present study believe that CPP-ACPF varnish is suitable for clinical use.

AFM is capable of producing images with atomic resolution with minimal sample preparation. This technique has been widely used to characterise the erosion of enamel and dentine. More recently, also AFM nanoindentation has also been applied to the study of enamel erosion.^2^ AFM microscopy was used in the present study to evaluate morphological changes on enamel after erosion with images of high contrast and resolution. The treatment of CPP-ACP was also found to facilitate the formation of a crystal layer, filling the interprism and partially covering the prisms, thus preventing acid attack.^21^ In line with our results, Poggio et al^21^ and Ceci et al^2^ also demonstrated that treatment with CPP-ACP paste to prevent dental erosion reduced the surface roughness measurements, as shown by AFM. According to our previous studies, CPP-ACPF showed lower surface roughness compared to NaF and fTCP. By contrast, all the tested varnishes showed a statistically significant decrease in surface roughness compared with negative control group but there was no statistically significant difference between treatment groups (p >0.05). The application of CCP-ACPF varnish results in the formation of a superficial homogeneous layer. After erosion with an acidic substance such as Coca Cola, the surface should appear much rougher. Enamel prism and enamel interprism structures became clearly visible on AFM images after the acid etching of smoothed natural enamel surfaces.

The use of calcium and phosphate products together with fluoride has been reported to have a synergistic effect^12,13^; therefore, a sodium fluoride varnish, a sodium fluoride varnish containing tricalcium phosphate, and a sodium fluoride varnish containing CPP-ACP were included among the treatment regimens that were tested.

Our results showed that fluoride varnish with CPP-ACP was found to be more resistant to enamel erosion compared to the other varnishes. Fluoride varnish with CPP-ACP provides additional fluoride along with calcium and phosphate ions for remineralisation. Previous studies have shown that CPP-ACP and CPP-ACFP varnishes can significantly increase hardness^18,30^ and decrease erosion^21,22,29^ of enamel softened by erosive substances. CPP-ACP nanocomplexes located on the enamel surface have been purported to buffer the activity of free calcium and phosphate ions, thereby maintaining a state of supersaturation with respect to tooth enamel, preventing enamel demineralisation and promoting remineralisation.^21^ The treatment of CPP-ACP was also found to facilitate the formation of a crystal layer, filling the interprism and partially covering the prisms, thus preventing acid attack.^21^ In line with our results, Poggio et al^21^ and Ceci et al^2^ also demonstrated that treatment with CPP-ACP paste to prevent dental wear reduced the surface roughness measurements, as shown by AFM. The study’s findings indicate that fTCP varnish offers greater protection against the challenge of enamel erosion than NaF varnish; however, it reveals less protection than CPP-ACPF varnish. It may be explained by the high release of calcium and inorganic phosphate ions from CPP-ACPF varnish or by the low solubility of tricalcium phosphate from fTCP varnish.^3,4^

## CONCLUSION

In this study, varnish forms of casein phosphopeptide amorphous calcium phosphate with fluoride were applied with different approaches of enamel erosion using natural saliva for simulating oral conditions. Our results suggest that the varnish combination of CPP-ACP with fluoride provides a higher remineralising potential than fluoride used alone. The CPP-ACPF varnish increased fluoride incorporation in subsurface enamel and substantially increased remineralisation of enamel surface than with fluoride varnish. This may explain that when fluoride ions come together with free calcium and phosphate ions, fluorapatite rapidly forms in the surface layer. The CPP-ACPF varnish can serve as an agent for treatment and also prevention of enamel erosion.
